# Efficient Degradation of Monoacylglycerols by an Engineered *Aspergillus oryzae* Lipase: Synergistic Effects of sfGFP Fusion and Rational Design

**DOI:** 10.3390/molecules31030398

**Published:** 2026-01-23

**Authors:** Yuqing Wang, Fang Liu, Yuxi Tian, Jiazhen Sun, Dawei Liu, Fei Li, Yaping Wang, Ben Rao

**Affiliations:** 1 National Biopesticide Engineering Technology Research Center, Hubei Biopesticide Engineering Research Center, Hubei Academy of Agricultural Sciences, Biopesticide Branch of Hubei Innovation Centre of Agricultural Science and Technology, Wuhan 430064, China; m15572831569@163.com (Y.W.); liufang@nberc.com (F.L.); tianyuxi@nberc.com (Y.T.); jiazhensun@hbaas.com (J.S.); david.liu@grandhoyo.com (D.L.); feili@hbaas.ac.cn (F.L.); 2 State Key Laboratory of Biocatalysis and Enzyme, Engineering Hubei Collaborative Innovation Center for Green Transformation of Bio-Resources, Hubei Key Laboratory of Industrial Biotechnology, Biology Faculty of Hubei University, Hubei University, Wuhan 430062, China; 3 Wuhan Grand Hoyo Co., Ltd., Wuhan 430079, China

**Keywords:** *Aspergillus oryzae*, thermal stability, sfGFP, catalytic activity

## Abstract

Monoacylglycerols (MAGs) are significant intermediate byproducts in the hydrolysis of oils and fats. The accumulation of MAGs not only reduces the quality and purity of the final products in biodiesel production and edible oil refining but also poses challenges for downstream separation processes. Therefore, the development of efficient biocatalysts for the specific MAG conversion is of great industrial importance. The lipase from *Aspergillus oryzae* (AOL) has shown potential for lipid modification; however, the wild-type enzyme (WT) suffers from poor solubility, tendency to aggregate, and low specific activity towards MAGs in aqueous systems, which severely restricts its practical application. In this study, a combinatorial protein engineering strategy was employed to overcome these limitations. We integrated fusion protein technology with rational design to enhance both the functional expression and catalytic efficiency of AOL. Firstly, the superfolder green fluorescent protein (sfGFP) was fused to the N-terminus of AOL. The results indicated that the sfGFP fusion tag significantly improved the solubility and stability of the enzyme, preventing the formation of inclusion bodies. The fusion protein sfGFP-AOL exhibited a MAG conversion rate of approximately 65%, confirming the positive impact of the fusion tag on enzyme developability. To further boost catalytic performance, site-directed mutagenesis was performed based on structural analysis. Among the variants, the mutant sfGFP-Y92Q emerged as the most potent candidate. In the MAG conversion, sfGFP-Y92Q achieved a conversion rate of 98%, which was not only significantly higher than that of sfGFP-AOL but also outperformed the widely used commercial immobilized lipase, Novozym 435 (~54%). Structural modeling and docking analysis revealed that the Y92Q mutation optimized the geometry of the active site. The substitution of Tyrosine with Glutamine at position 92 likely enlarged the substrate-binding pocket and altered the local electrostatic environment, thereby relieving steric hindrance and facilitating the access of the bulky MAG substrate to the catalytic center. In conclusion, this work demonstrates that the synergistic application of sfGFP fusion and rational point mutation (Y92Q) can dramatically transform the catalytic properties of AOL. The engineered sfGFP-Y92Q variant serves as a robust and highly efficient biocatalyst for MAG degradation. Its superior performance compared to commercial standards suggests immense potential for cost-effective applications in the bio-manufacturing of high-purity fatty acids and biodiesel, offering a greener alternative to traditional chemical processes.

## 1. Introduction

Lipid hydrolysis constitutes a core unit operation within the oleochemical industry, finding extensive applications in critical sectors such as the preparation of high-purity fatty acids, the valorization of oil refining byproducts, the treatment of waste cooking oil, and the purification of crude glycerol from biodiesel production [1,2]. In these complex reaction matrices, the primary objective is typically the complete conversion of triacyl-glycerols (TAGs) and their partial hydrolysis products into high-value free fatty acids (FFAs) and glycerol. However, monoacylglycerols (MAGs), serving as intermediate products, often act as a critical bottleneck that limits both conversion rates and product purity [3,4]. Due to the pronounced amphiphilic nature and emulsifying properties of MAGs, their residual accumulation in reaction systems not only impedes effective oil-water phase separation—thereby complicating downstream purification processes [5,6]—but also precludes the attainment of theoretical maximum yields for oleochemical products such as fatty acids.

Although the traditional Colgate-Emery process, operating under high temperature and pressure, demonstrates high efficiency, its energy-intensive nature and association with undesirable side reactions—such as product darkening and oxidative polymerization—severely restrict its applicability for heat-sensitive feedstocks [7]. Consequently, enzymatic hydrolysis has garnered increasing attention as a green alternative, distinguished by its mild reaction conditions and benign environmental profile [8,9]. Nevertheless, most commercially available lipases predominantly exhibit high activity towards the *sn-1*,*3* ester bonds of TAGs [10]. Their catalytic efficiency towards MAGs (particularly *sn-2* MAGs) is frequently compromised by steric hindrance or product inhibition, resulting in a kinetic pattern characterized by rapid initial hydrolysis followed by stagnation, which hinders complete substrate degradation. Therefore, the development and investigation of lipases possessing high specificity and catalytic activity towards monoacylglycerols is of significant academic and industrial importance [11,12]. Such biocatalysts are essential for addressing the ubiquitous emulsification and separation challenges in the oleochemical industry, achieving the deep valorization of waste oil resources, and advancing the development of green bio-manufacturing processes.

*Aspergillus oryzae* lipase (AOL), a potent biocatalyst originating from filamentous fungi, is highly regarded in the biotechnology sector for its exceptional enzymatic characteristics [13,14,15]. Distinguished by superior regioselectivity (typically favoring *sn-1,3* positions) and stereospecificity, AOL is capable of efficiently catalyzing a diverse array of reactions—including hydrolysis, esterification, and transesterification—under mild conditions [16,17]. Furthermore, the enzyme exhibits broad adaptability towards fatty acid substrates of varying chain lengths and maintains robust activity and stability even within organic solvent systems. These distinctive features endow AOL with immense industrial value, rendering it an ideal biocatalyst for applications such as lipid modification, chiral resolution, and biofuel production.

The *Escherichia coli* (*E. coli*) expression system is currently the most widely used platform for recombinant protein production, owing to its significant advantages such as a well-characterized genetic background, short culture cycles, and low costs [18]. However, the overexpression of exogenous proteins in this system often leads to the aggregation of insoluble inclusion bodies due to folding rate mismatches or intracellular environmental limitations, which represents a major bottleneck restricting its application. To address this challenge, sfGFP (superfolder GFP), a deeply evolved fluorescent marker, offers an excellent solution. With its optimized folding kinetics, sfGFP is capable of rapid folding and generating stable fluorescent signals within complex intracellular environments. More importantly, when utilized as a fusion tag, it exerts a ‘chaperone-like’ effect that assists in the correct folding of its fusion partner proteins, thereby significantly inhibiting inclusion body formation and enhancing protein solubility and expression efficiency. For example, a study by Xingyu Yan et al. confirmed that the use of the sfGFP tag successfully overcame the folding obstacles of novel unspecific peroxidases (UPOs) in *E. coli*, achieving efficient soluble expression and extracellular secretion of the enzyme.

Enzyme molecular engineering serves as a pivotal technology for enhancing the catalytic performance and industrial adaptability of natural enzymes, holding significant value in sectors such as biomedicine and bioenergy [19]. To address limitations like poor stability, current strategies primarily encompass irrational, semi-rational, and rational design. Irrational design involves screening random mutants by mimicking natural evolution, while semi-rational design integrates structural analysis with directed evolution to optimize performance. Rational design, conversely, executes precise modifications based on structural mechanisms, characterized by high screening efficiency and specificity [20]. The synergistic advancement of these approaches has significantly transcended the constraints of enzymatic properties, establishing a robust foundation for the customized engineering and industrial application of enzyme preparations.

This study presents a comprehensive solution to the challenges of poor solubility and low catalytic efficiency associated with lipase AOL expression in *Escherichia coli*. First, by utilizing sfGFP fusion tag technology, efficient soluble expression of AOL was achieved, increasing purification yields by over 40%. Second, through homology modeling and rational design, key mutants Y92Q and Y92I were identified, exhibiting a specific activity increase of more than 3.5-fold compared to the control. Crucially, these mutants demonstrated exceptional performance in the deep hydrolysis of monoacylglycerols (MAGs), effectively overcoming the kinetic bottleneck typically caused by MAG accumulation in late-stage lipid hydrolysis. Mechanistic analysis revealed that these mutations reduced steric hindrance and optimized the hydrogen bond network, thereby facilitating the access of MAG substrates to the catalytic center and significantly enhancing both catalytic efficiency and thermostability. Notably, the Y92Q mutant showed a 1.8-fold extension in half-life at 50 °C compared to the wild type. These findings provide an efficient biocatalyst for eliminating MAG residues to achieve high-purity glyceride synthesis and establish a general strategy for the industrial engineering of microbial lipases.

## 2. Results and Discussion

### 2.1. Soluble Expression of sfGFP-AOL and AOL-sfGFP Fusion Proteins

In this study, although the individual expression of AOL yielded a specific band in the whole-cell lysate consistent with the theoretical molecular weight (35 kDa), SDS-PAGE analysis revealed that the protein existed primarily as inactive inclusion bodies. Furthermore, no enzymatic activity was detected even after denaturation and refolding. To address this folding defect, sfGFP was subsequently introduced as a fusion tag at both the N-terminus and C-terminus of AOL, aiming to enhance protein folding efficiency and achieve soluble expression.

**Figure 1:**
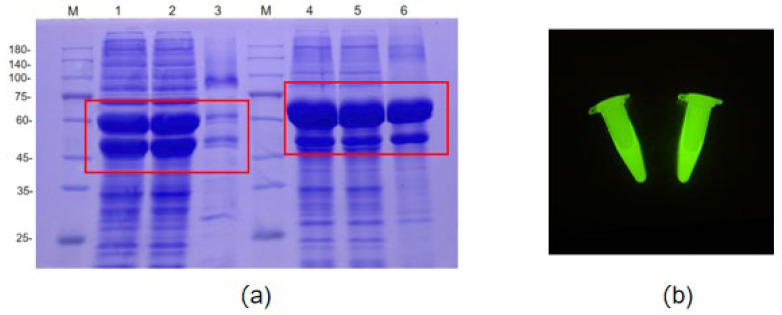
Analysis of the soluble expression of sfGFP-AOL and AOL-sfGFP fusion proteins. (**a**) SDS-PAGE analysis of protein solubility. Lane M: 180 kDa Protein Marker; Lane 1: Whole cell lysate of sfGFP-AOL; Lane 2: Supernatant of sfGFP-AOL; Lane 3: Pellet of sfGFP-AOL; Lane 4: Whole cell lysate of AOL-sfGFP; Lane 5: Supernatant of AOL-sfGFP; Lane 6: Pellet of AOL-sfGFP. The red rectangles indicate the target fusion protein bands. (**b**) Fluorescence observation of the cell lysate supernatants under blue light excitation. Left: AOL-sfGFP; Right: sfGFP-AOL.

According to the SDS-PAGE analysis, a specific band consistent with the predicted molecular weight was observed in the lysate supernatant (Figure 1a), which also exhibited distinct green fluorescence under blue light excitation (Figure 1b). These findings confirm that fusing the sfGFP tag to either the N- or C-terminus of the target gene effectively promotes the soluble expression of the fusion protein. The comparison between the supernatant and pellet fractions indicates that the sfGFP moiety significantly enhanced protein folding efficiency, facilitating the correct folding of AOL, which had previously aggregated as inclusion bodies due to misfolding. This suggests that sfGFP may mediate soluble expression via a mechanism distinct from that of traditional signal peptides. In conclusion, the soluble expression of the AOL protein was successfully achieved through this sfGFP-mediated strategy.

### 2.2. Activity Assay of the Target Protein

*Aspergillus oryzae* lipase (AOL) is typically characterized as a partial glyceride lipase exhibiting hydrolytic activity towards both monoacylglycerols (MAG) and diacylglycerols (DAG). To evaluate the catalytic performance of the sfGFP-AOL and AOL-sfGFP fusion proteins, a lipid mixture was employed as the substrate, and the reaction products were analyzed using both thin-layer chromatography (TLC) for qualitative assessment and High-Performance Liquid Chromatography (HPLC) for quantitative verification.

**Figure 2:**
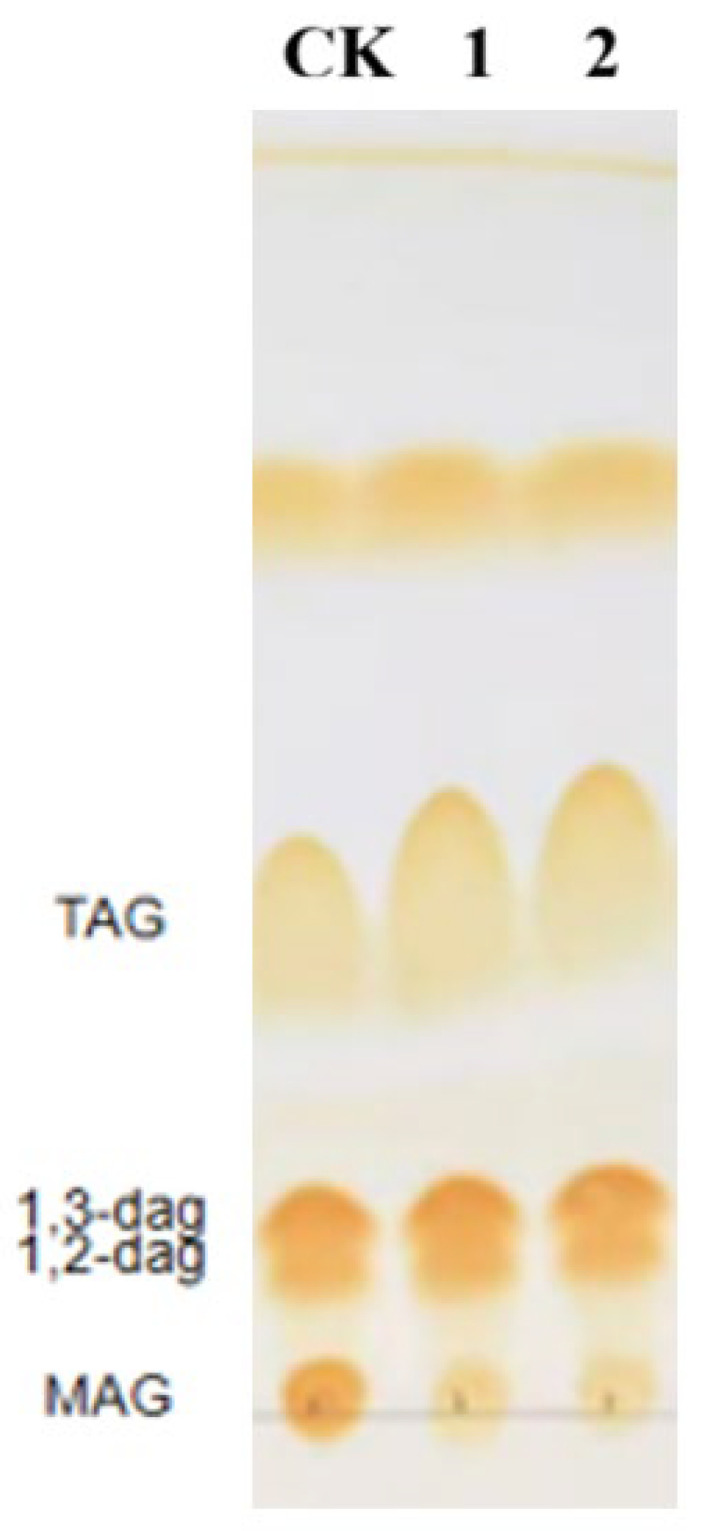
Qualitative analysis of the hydrolytic activity of sfGFP-AOL (lane 1) and AOL-sfGFP (lane 2) by thin-layer chromatography.

Consistent with the TLC results (Figure 2), which showed a marked reduction in the intensity of MAG spots compared to the negative control, quantitative HPLC analysis revealed a substantial MAG conversion rate of approximately 50%. In sharp contrast, the hydrolytic efficiency towards poly-acylated substrates was minimal, with conversion rates of only 5% for DAG and 3% for TAG (triacylglycerols). These results demonstrate that the fusion proteins exhibit a distinct substrate preference for MAG within this reaction system, showing negligible activity towards DAG and TAG. Consequently, based on this pronounced specificity, subsequent experiments were designed to focus specifically on optimizing the hydrolytic activity of the lipase towards MAG.

To evaluate the potential influence of the sfGFP fusion tag on the catalytic performance of AOL, we determined the kinetic parameters (Km and kcat) of both sfGFP–AOL and AOL–sfGFP using the target substrate, 1-monoolein (MAG). While both fusion constructs retained measurable activity, the N-terminal fusion (sfGFP–AOL) exhibited a significantly higher catalytic efficiency (kcat/Km) compared to the C-terminal fusion (AOL–sfGFP). Specifically, the turnover numbers (kcat) were determined to be 12 s^−1^ for sfGFP–AOL and 5 s^−1^ for AOL–sfGFP, with corresponding apparent values of 0.5 mM and 1.2 mM, respectively. These results indicate that although the enzyme remains active in both configurations, the positioning of the sfGFP tag modulates substrate accessibility and turnover at the oil–water interface, with the N-terminal placement being structurally more favorable. Consequently, sfGFP–AOL was selected for all subsequent studies owing to its superior kinetic profile.

Complementing the kinetic data, qualitative analysis via thin-layer chromatography (TLC) provided critical insights into the substrate specificity. A key finding is the pronounced hydrolytic capability of the fusion enzymes towards monoacylglycerols (MAG) versus diacylglycerols (DAG). As evidenced by the TLC profiles, MAG spots were significantly diminished, whereas substantial DAG substrate remained unhydrolyzed. This distinct preference suggests that the introduction of the sfGFP moiety creates a specific steric environment around the active site. We postulate that the smaller MAG molecule accesses the catalytic center with minimal hindrance, thereby facilitating rapid hydrolysis. Conversely, the bulkier DAG molecule likely faces greater resistance entering the active pocket in the presence of the fusion tag. Crucially, this steric hypothesis aligns with our kinetic observations: the N-terminal fusion likely imposes less steric constraint than the C-terminal fusion, resulting in the observed higher and lower. The ability of sfGFP-AOL to preferentially and deeply hydrolyze MAGs is particularly advantageous for industrial applications, where MAG accumulation often acts as a bottleneck. Thus, the sfGFP-AOL fusion protein presents a potent solution for overcoming kinetic limitations in lipid hydrolysis processes.

### 2.3. Structural Analysis, Alanine Scanning, and Virtual Saturation Mutagenesis of AOL

**Figure 3:**
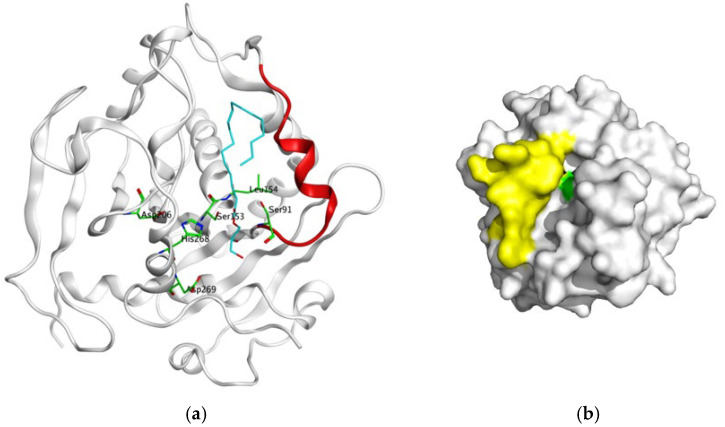
(**a**): Superposition of the AOL (open-lid) and wild-type AOL (closed-lid) structures; (**b**): Surface representation of the AOL open-lid conformation.

The surface representation of the AOL open-lid conformation was visualized using PyMOL version 3.1.6 (Figure 3a) and superimposed onto the closed-lid structure (Figure 3b). Structural alignment reveals that while the overall architecture is conserved, distinct conformational differences are evident in the lid domain (yellow for open, cyan for closed) and the position of the key catalytic residue S153 (green).

The open-lid conformation creates a larger access channel, facilitating substrate entry into the active site. In contrast, the closed-lid conformation likely forms a sealed environment through hydrophobic interactions or hydrogen bonding with surrounding residues, preventing substrate access. Functionally, the closed state may protect the active site during catalysis and assist in product release via conformational changes. Analyzing these structural dynamics provides insights into the mutant’s catalytic mechanism. Consequently, future rational design strategies will focus on introducing mutations to optimize lid dynamics, thereby enhancing catalytic efficiency.

Molecular docking of AOL with the substrate 1 monoolein was first performed. To enhance enzymatic activity, it is essential to improve the affinity between the enzyme and the substrate. This requires rational mutation of amino acids within the active pocket to either reduce steric hindrance for substrate entry or increase specific contacts between the protein and the substrate. Based on the docked complex, computational alanine scanning was conducted using the Rosetta software suite. Residues within a 5 Å radius of the ligand were selected for the scan, excluding the catalytic triad. Each selected residue was virtually mutated to alanine (Ala). Subsequently, side-chain repacking and energy minimization were performed on the mutant structures to resolve any steric clashes or unfavorable conformations introduced by the mutations. Finally, the change in binding free energy ($\Delta \Delta G_{binding}$) was calculated to evaluate the contribution of each residue to the stability of the enzyme-substrate complex.

**Figure 4:**
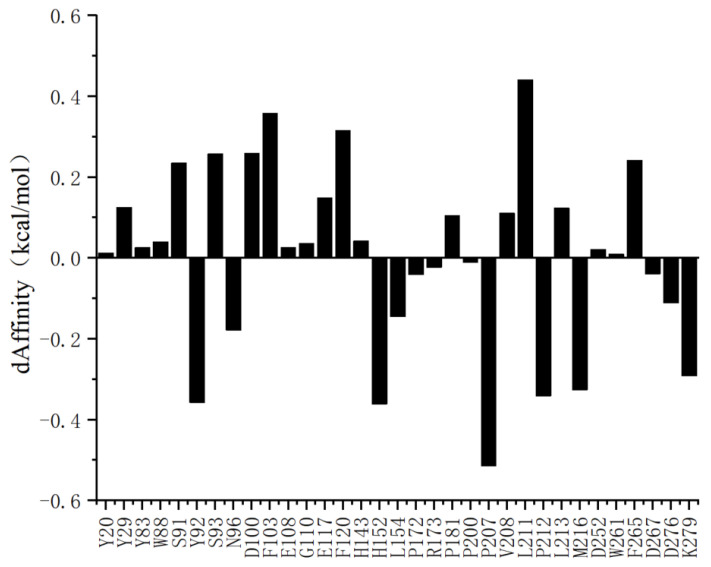
Virtual alanine scanning results of the AOL enzyme.

A threshold of ±0.05 kcal/mol was established; residues exhibiting free energy changes exceeding this limit were considered to have a significant impact on the AOL-substrate interaction. The results (Figure 4) indicated substantial variations in binding free energy for the following residues: Y92, N96, H152, L154, P207, P212, M216, D276 and K279. Based on these findings, these residues were selected as candidates for the subsequent round of saturation mutagenesis.

**Figure 5:**
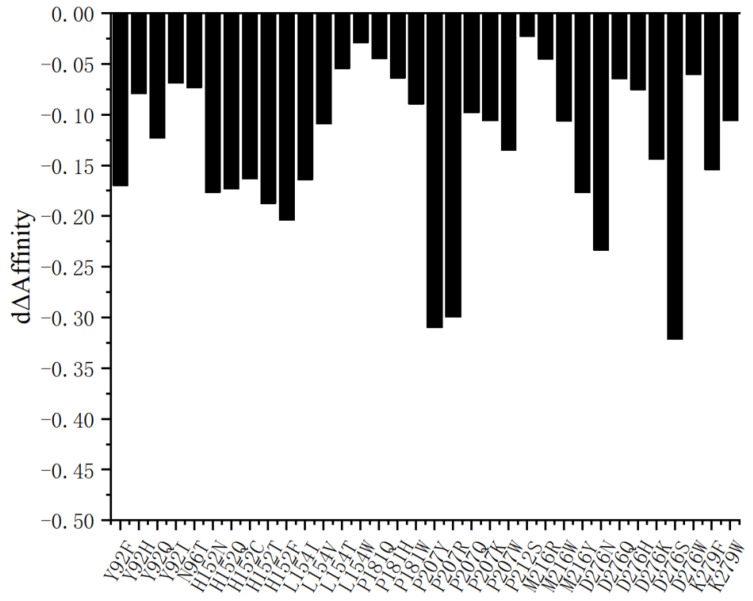
Virtual saturation mutagenesis results of the AOL enzyme.

Based on the key sites identified via alanine scanning, virtual saturation mutagenesis was performed to substitute these residues with the remaining 19 natural amino acids. Following a systematic analysis of computational data, mutations exhibiting significant shifts in binding free energy (dΔAffinity) were selected (Figure 5). Consequently, a total of 59 candidate saturation mutants were designated for experimental construction. In the subsequent experimental phase, these candidates will undergo heterologous expression, purification, and enzymatic activity assays. These procedures aim to validate the accuracy of the in silico design and to further explore effective strategies for enhancing enzymatic activity.

Here, a rational design strategy for the AOL enzyme was established by integrating molecular docking, computational alanine scanning, and virtual saturation mutagenesis. Our findings demonstrate that nine residues located within a 5 Å radius of the active pocket—including Y92 and P207—serve as critical determinants of enzyme-substrate interactions. The identification of 59 candidate mutants exhibiting significant shifts in binding free energy suggests that optimizing these specific loci can effectively mitigate steric hindrance or augment specific contacts. Crucially, this computation-driven methodology substantially narrowed the screening scope, thereby circumventing the inefficiencies inherent in traditional random mutagenesis. Subsequent experimental validation of these candidates will not only corroborate the accuracy of the in silico design but also provide a fundamental basis for elucidating the catalytic mechanism of AOL and developing high-performance industrial biocatalysts.

### 2.4. Quantitative Assay of Mutant Enzymatic Activity

**Figure 6:**
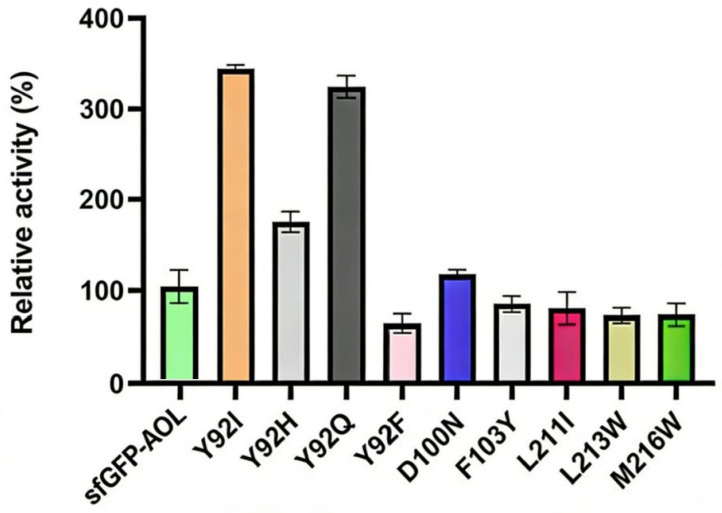
Comparison of specific activities between sfGFP-AOL and mutant variants.

Following a screening process utilizing Thin Layer Chromatography (TLC) and RP-HPLC-ELSD, ten mutants exhibiting hydrolytic activity toward monoacylglycerols were identified. The candidates were subsequently expressed, purified, and subjected to kinetic characterization using the target substrate, 1-monoolein (MAG) (Figure 6). Notably, two mutants, sfGFP-AOL-Y92Q and sfGFP-AOL-Y92I, demonstrated significantly enhanced enzymatic properties. Their catalytic efficiencies (kcat/Km) were determined to be 42.3 s^−1^mM^−1^ and 46.7 s^−1^mM^−1^, respectively. These values represent a substantial improvement of 3.5-fold and 3.9-fold compared to the parental sfGFP-AOL, which exhibited a baseline efficiency of 12 s^−1^mM^−1^.

The successful isolation of ten active mutants validates the efficacy of the combined TLC and RP-HPLC-ELSD screening strategy. However, the most striking finding is the superior catalytic performance of variants sfGFP-AOL-Y92Q and sfGFP-AOL-Y92I. The observed 3.5- to 3.9-fold increase in specific activity suggests that modifying residue Y92 significantly alleviates catalytic constraints present in the wild-type enzyme. This enhancement implies that the substitution of Tyrosine with Glutamine or Isoleucine may optimize the active site geometry, thereby improving substrate accessibility or stabilizing the transition state during monoacylglycerol hydrolysis. These results not only validate the predictive accuracy of the initial in silico saturation mutagenesis but also establish Y92Q and Y92I as robust candidates for industrial applications requiring high-efficiency biocatalysts.

### 2.5. Characterization of Enzymatic Properties of the Two Mutants

In addition to catalytic activity, environmental adaptability and thermal stability are critical indicators determining the suitability of enzymes for industrial applications. To determine whether mutations at key residues altered the optimal reaction conditions or enhanced structural stability, this study systematically characterized the enzymatic profiles of mutants sfGFP-AOL-Y92Q and sfGFP-AOL-Y92I across various temperature and pH gradients and quantitatively assessed their thermal tolerance.

**Figure 7:**
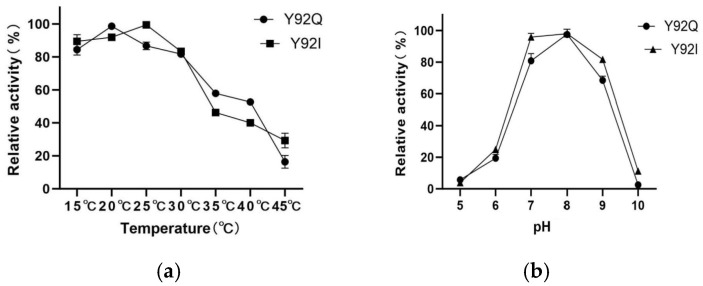
(**a**) Effect of Temperature on Mutant Enzyme Activity; (**b**) Effect of pH on Mutant Enzyme Activity.

Both mutants retained substantial activity at low-to-medium temperatures within the tested range of 15 °C to 45 °C. The optimal reaction temperatures for sfGFP-Y92Q and sfGFP-AOL-Y92I were identified as 20 °C and 25 °C, respectively (Figure 7a). Regarding pH sensitivity (tested from pH 5.0 to 10.0), both mutants exhibited a similar trend: activity increased between pH 5.0 and 7.0, peaking at an optimum of pH 8.0 (Figure 7b). These results indicate that the mutations did not significantly alter the optimal pH range compared to the wild type.

To assess thermal stability, the enzymes were incubated at 50 °C for durations ranging from 0 to 3 h. Samples were immediately quenched in an ice bath prior to residual activity measurement. The data demonstrated that both mutants possessed significantly enhanced thermostability compared to the wild type. Notably, mutant sfGFP-Y92Q exhibited the most robust stability, retaining 62.4% ± 1.7% of its residual activity after 3 h of incubation. Its thermal half-life ($t_{1/2}$) was extended by 2.3-fold compared to the wild type. While mutant Y92I also showed improved stability (with a 1.8-fold increase in $t_{1/2}$), the magnitude of this enhancement was significantly lower than that of Y92Q (p<0.01).

**Figure 8:**
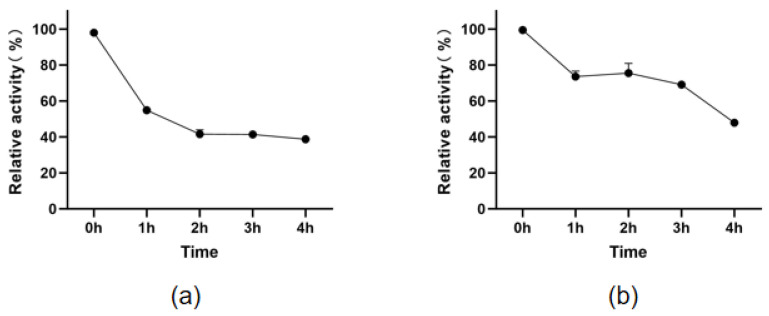
Comparison of thermal stability between (**a**) sfGFP-Y92I and (**b**) sfGFP-Y92Q.

The evaluation of mutants sfGFP-Y92Q and sfGFP-Y92I reveals significant insights into their enzymatic properties, crucial for potential industrial applications. The retention of substantial activity at low-to-medium temperatures, with optimal temperatures of 20 °C and 25 °C, respectively, suggests that these mutants are well-suited for processes requiring mild conditions. Importantly, the thermal stability assessments demonstrate (Figure 8) that both mutants exhibit enhanced thermostability, with sfGFP-Y92Q showing exceptional resilience by retaining over 62% of its activity after 3 h at 50 °C. The significant increase in thermal half-life ($t_{1/2}$) for sfGFP-Y92Q (2.3-fold) compared to the wild type emphasizes its potential for industrial applications where elevated temperatures are common. These findings underscore the potential of these mutants as viable candidates for biocatalytic applications in various industrial processes, particularly those requiring thermal resilience.

**Figure 9:**
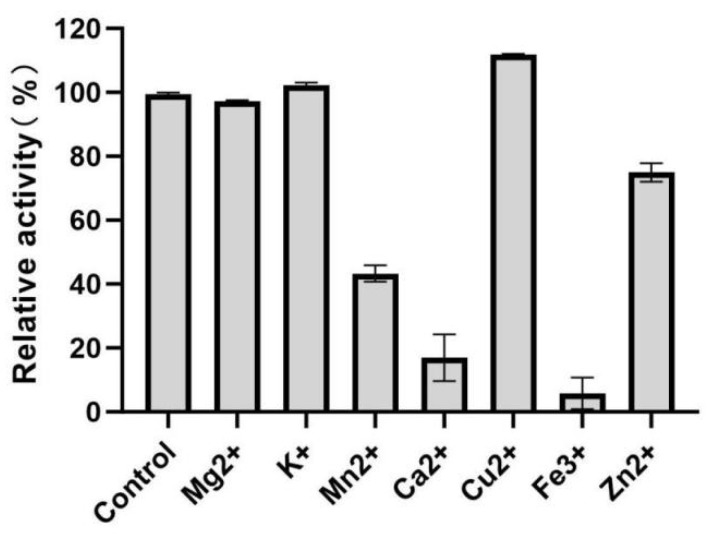
Effect of various metal ions on the relative activity of the engineered lipase.

Given that the mutant sfGFP-AOL-Y92Q exhibits the best stability, this study focuses on it as the subject of investigation. Eight metal ions were selected, and their effects on enzyme activity were measured using the p-nitrophenol assay at 20 °C and pH 8.0, with a concentration of 5 mM for each metal ion (Figure 9). The results indicated that among the eight metal ions, Cu^2+^ and K^+^ slightly enhanced the relative activity of the enzyme. This effect may be attributed to their ability to provide a favorable charge environment, thereby stabilizing the active conformation of the enzyme or promoting substrate binding efficiency. In contrast, Mn^2+^, Ca^2+^, Fe^3+^, and Zn^2+^ exhibited varying degrees of inhibitory effects on enzyme activity, likely due to alterations in the enzyme’s charge distribution or interference with the structure of the active site, which suppressed the catalytic ability of the enzyme. Future studies could consider adjusting the concentrations of metal ions to further investigate the changes in enzyme activity under different concentration conditions.

### 2.6. Molecular Dynamics Simulation and RMSD Analysis

**Figure 10:**
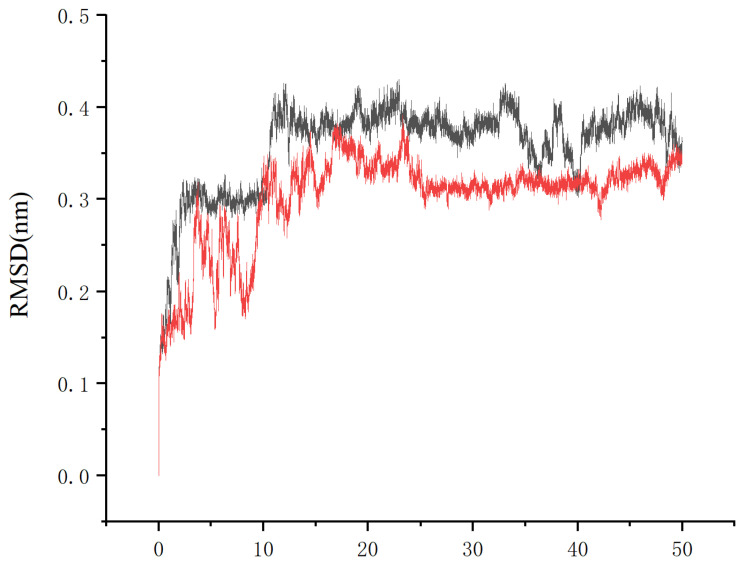
Root Mean Square Deviation (RMSD) analysis of sGFP-AOL (Black line) and Y92Q (Red line) mutant during 50 ns molecular dynamics simulation.

To investigate the impact of the mutation on structural stability, molecular dynamics (MD) simulations were performed for 50 ns at 300 K using the Amber 22 software package for both sGFP-AOL and the mutant Y92Q. The Root Mean Square Deviation (RMSD) was calculated to analyze the structural dynamic differences between the two (Figure 10).

The trajectory analysis revealed that the RMSD of sGFP-AOL stabilized at 0.40 ± 0.04 nm, exhibiting significant fluctuations (maximum difference of 0.10 nm). In contrast, the RMSD of the Y92Q mutant (red line) stabilized at 0.32 ± 0.04 nm, with a fluctuation amplitude 20% lower than that of the sGFP-AOL, and no anomalous peaks were observed. Y92Q demonstrated significantly better structural convergence compared to sGFP-AOL, suggesting that the mutation enhances the overall structural rigidity of the protein. These computational results are highly consistent with the experimental observations of improved thermal stability and enzymatic activity in the Y92Q mutant. The more stable conformation of Y92Q likely facilitates the maintenance of the active site geometry, thereby potentially enhancing substrate affinity and sustaining catalysis, which ultimately contributes to its superior enzymatic activity and thermostability.

### 2.7. Assessment of Monoacylglycerol Hydrolysis by sfGFP-AOL-Y92Q

To evaluate the hydrolytic activity of sfGFP-Y92Q towards monoacylglycerols (MAG), the enzyme was mixed with the MAG-containing substrate at a mass ratio of 1:30. The substrate contains 50% MAG with the remaining 50% composed of diacylglycerols (DAGs) and triglycerides (TAGs). The reaction was conducted at 20 °C in a system buffered with PBS (pH 8.0), maintaining a water-to-oil ratio of 4:1. Upon completion of the reaction, the mixture was centrifuged, and the upper oil layer was collected. The samples were subsequently analyzed using RP-HPLC-ELSD to quantify the extent of hydrolysis. Each reaction was performed in triplicate to ensure reproducibility. The conversion of MAG was calculated using the formula:

Conversion Rate (%) = MAG consumed/Initial MAG × 100

**Figure 11:**
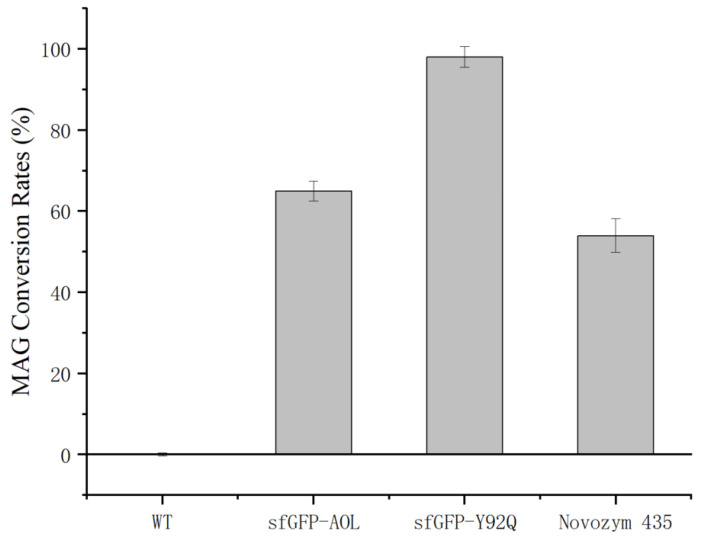
Comparison of MAG Conversion Rates catalyzed by different lipase variants and the commercial enzyme Novozym 435.

The hydrolytic efficacy of the different enzyme preparations towards MAG was evaluated and compared, as illustrated in Figure 11. To ensure a rigorous comparison between the free enzymes and the immobilized commercial control (Novozym 435), dosages were normalized based on active protein mass, assuming a protein loading of 10% (*w*/*w*) for the immobilized catalyst. Under these conditions, the mutant sfGFP-Y92Q exhibited superior catalytic performance, achieving a MAG conversion rate of approximately 98%. This was significantly higher than both the fusion protein sfGFP-AOL (65%) and the commercial benchmark Novozym 435 (54%) (p<0.05). In contrast, the wild-type (WT) showed negligible hydrolytic activity. One-way ANOVA analysis (SPSS 27.0) confirmed that the observed hierarchy of activity (sfGFP-Y92Q > sfGFP-AOL > Novozym 435 > WT) was statistically significant.

These results underscore the synergistic impact of fusion technology and rational design in optimizing biocatalytic performance. The superior activity of sfGFP-AOL compared to the wild-type (WT) enzyme suggests that the sfGFP fusion partner plays a critical role in stabilizing the hydrophobic AOL domain, likely by preventing the aggregation and inactivation observed in the WT control. Furthermore, the exceptional performance of the sfGFP-Y92Q variant highlights the efficacy of active-site engineering; the Y92Q mutation appears to optimize the substrate-binding pocket—potentially by altering the local amphiphilic environment or relieving steric hindrance—thereby facilitating more efficient catalysis of MAG substrates.

While the inclusion of the sfGFP moiety increases the molecular weight of the biocatalyst, raising questions regarding atom economy, its retention represents a strategic trade-off for industrial viability. Although the engineered 3C site allows for tag removal, such a step would necessitate complex downstream processing (proteolysis and re-purification), significantly increasing operational costs. Moreover, given the intrinsic hydrophobicity of AOL, tag removal poses a substantial risk of protein aggregation and loss of function. Therefore, the direct utilization of the intact sfGFP-Y92Q fusion protein prioritizes high soluble yields and process simplicity over mass-normalized specific activity. This ‘one-step’ preparation strategy, combined with the capability for real-time monitoring of reactor loading via fluorescence, establishes sfGFP-Y92Q as a robust, cost-effective candidate for green biomanufacturing applications, particularly in the efficient removal of monoacylglycerol impurities for high-quality biodiesel production.

## 3. Materials and Methods

### 3.1. Strains and Reagents

*Escherichia coli* DH5α were preserved in our laboratory. Protein molecular weight standards (catalog number: 3450), ligases (catalog number: 2011A), Taq polymerase (catalog number: RR001Q), and nucleic acid molecular weight standards (catalog number: 3408) were purchased from TaKaRa company (Kusatsu, Japan). All other reagents were of domestic analytical grade.

### 3.2. Primer Design and PCR Amplification

PCR amplification was performed using PrimeSTAR Max Premix (TaKaRa, Japan, Kusatsu). The reaction mixture (10.5 μL) consisted of 1 μL of template DNA, 0.3 μL each of forward and reverse primers, 5 μL of Premix, and 3.9 μL of ddH_2_O. The thermal cycling protocol included an initial denaturation at 95 °C for 5 min, followed by 28 cycles of denaturation at 95 °C for 20 s, annealing at 55–65 °C (optimized for specific primers) for 20 s, and extension at 72 °C (duration adjusted based on a synthesis rate of 6 kb/min). A final extension was performed at 72 °C for 5 min, followed by a hold at 12 °C. The primer sequences are listed in Supplementary Materials File.

### 3.3. Recombinant Plasmids Construction

The AOL gene (GenBank Accession No. BAA12912.1), originating from *Aspergillus oryzae*, was selected for this study. The gene sequence was codon-optimized, and a site-directed mutation was introduced to replace Valine at position 269 with Aspartic acid (V269D). The full-length gene fragments were synthesized using overlap extension PCR ((TaKaRa, Japan, Kusatsu)) and subsequently cloned into the pET-23a vector backbone via inverse PCR. Three recombinant expression vectors were constructed: pET-23a-AOL, pET23a-sfGFP-3c-AOL, and pET23a-AOL-3c-sfGFP. The fidelity of all recombinant plasmids was confirmed by DNA sequencing performed by Wuhan GeneCreate Biological Engineering Co., Ltd. (Wuhan, China)

### 3.4. Recombinant Strains Construction and Protein Expression

To express the AOL protein, the recombinant plasmid pET23a-AOL was transformed into *E. coli* BL21 (DE3) competent cells. The transformants were plated onto Luria–Bertani (LB) agar plates containing ampicillin and incubated overnight at 37 °C. A single colony was picked and inoculated into fresh LB liquid medium supplemented with ampicillin, then incubated at 37 °C with shaking until the optical density at 600 nm (OD_600_) reached approximately 0.6. Protein expression was induced by the addition of IPTG, and the culture was further incubated at 18 °C with shaking at 220 rpm for 16 h. Subsequently, the cells were harvested and disrupted using a high-pressure homogenizer. The whole cell lysate was centrifuged to separate the supernatant and the pellet. Samples from the whole lysate, supernatant, and pellet were analyzed by SDS-PAGE to verify the expression and solubility of the target protein.

### 3.5. Activity Evaluation of AOL Using TLC and HPLC

Glyceride composition was analyzed using Thin Layer Chromatography (TLC). HSGF254 silica gel plates were used as the stationary phase, and a mixture of n-hexane, diethyl ether, and acetic acid (70:30:1, *v*/*v*/*v*) was used as the mobile phase. For sample preparation, 1 μL of glyceride was dissolved in 50 μL of the mobile phase, and 4.5 μL of the mixture was spotted onto the plate, 1 cm from the bottom edge. After the spots were dried, the plate was developed in a chromatography tank. Upon completion, the plate was air-dried and visualized by exposure to iodine vapor. The glycerides were qualitatively identified by comparing their retention factors (Rf) with those of standards. Here TLC was used for qualitative/semi-quantitative screening; quantitative conversion was determined by RP-HPLC-ELSD.

The reaction products were analyzed using a Reverse Phase High-Performance Liquid Chromatography system coupled with an Evaporative Light Scattering Detector (RP-HPLC-ELSD, Thermo Fisher Scientific). Separation was performed on a GL Science Inertsil ODS-3 column (4.6 × 150 mm, 5 μm) maintained at 30 °C. The mobile phase consisted of acetonitrile with 0.1% (*v*/*v*) acetic acid (Solvent A) and isopropanol (Solvent B) at a flow rate of 0.8 mL/min. The elution gradient was applied as follows: 0–2 min, 30% A; 2–15 min, 30–40% A; 15–22 min, 60% A; 22–25 min, 60–30% A; and 25–30 min, 30% A for equilibration. The ELSD drift tube temperature was set to 95 °C, with a nitrogen carrier gas flow of 2.0 L/min. For analysis, 1 μL of the reaction product was mixed with 800 μL of isopropanol and 800 μL of acetonitrile. A 20 μL aliquot of the prepared sample was injected, and data were analyzed using the Chromeleon 6.8 workstation.

### 3.6. Protein Stability Assay

(1) Protein Structure Prediction and Modeling

The 3D structure of the mutant AOL was predicted using the AlphaFold 3 system. The amino acid sequence was used as the input for the deep learning-based ab initio folding simulation.

(2) Molecular Docking and Complex Modeling

To elucidate the catalytic mechanism, molecular docking with the substrate (1,3-dilinolein) was performed using AutoDock 4.2. The 3D structures of the ligands were retrieved using ChemDraw 20.0 and optimized using OpenBabel. The receptor protein was pre-processed in PyMOL to remove water molecules and ions, followed by the addition of polar hydrogens and Gasteiger charges using AutoDockTools to generate PDBQT files. The center of the grid box was defined based on the active site using the PyMOL plugin center_of_mass.py. A search algorithm was employed to explore ligand conformations, and the conformation with the lowest binding energy was selected as the template.

(3) Identification of Mutation Hotspots (Alanine Scanning)

Based on the docked complex, residues within 5 Å of the substrate were selected as candidate sites. Initial screening was performed using the Residue Scanning Calculations plugin in PyMOL. Subsequently, the Rosetta software suite was employed for precise evaluation. The change in Gibbs free energy (ΔΔG) was calculated for each mutant. ΔΔG is defined as the difference in total system energy between the mutant and the wild type ($\Delta G_{mutant}-\Delta G_{wild-type}$). Negative values (ΔΔG<0) indicate improved stability or binding affinity, while positive values indicate a decrease in stability.

(4) Simulated Saturation Mutagenesis and Screening

Key residues identified by alanine scanning were subjected to virtual saturation mutagenesis using Rosetta. Each target site was mutated to the other 19 natural amino acids using Rosetta’s mutagenesis protocols, followed by side-chain relaxation. The relative change in free energy (ΔΔG in Rosetta Energy Units, REU) was calculated for each mutant. Mutants exhibiting an energy change greater than ±0.1 REU (or kcal/mol) were considered to significantly impact enzyme-substrate interactions and were prioritized for experimental validation.

### 3.7. Characteristics of the Enzyme AOL

To characterize the biochemical properties of the recombinant lipase AOL, enzymatic activity was assayed using 1-monoolein (MAG) as the substrate. The primary metric for characterization across all experiments was the catalytic efficiency (kcat/Km), with all assays performed in triplicate to ensure reliability.

For the standard assay, the enzyme solution was diluted to an appropriate concentration with 50 mM Tris-HCl buffer (pH 8.5). The reaction mixture contained the diluted enzyme and MAG substrate dissolved in the same buffer. The mixture was pre-incubated at 40 °C for 5 min to achieve temperature equilibrium before initiating the reaction. The reaction was allowed to proceed for a duration within the linear range of product formation and was terminated by heat inactivation or the addition of an organic solvent, such as acetonitrile. The hydrolysis products were quantified using a suitable analytical method, such as high-performance liquid chromatography (HPLC) or a coupled enzymatic assay, to determine the initial reaction velocity (v0) under different substrate concentrations.

A series of reactions with varying MAG concentrations were performed to deter mine the Michaelis constant (Km) and the turnover number (kcat). The kinetic parameters Km and Vmax were derived by fitting the initial velocity data to the Michaelis-Menten equation using non-linear regression. The kcat value was then calculated from the Vmax and the molar concentration of the active enzyme. The catalytic efficiency (kcat/Km) was computed as the ratio of kcat to Km.

To evaluate thermostability, the enzyme was pre-incubated at different temperatures (35, 40, 45, and 50 °C) for varying durations. After pre-incubation, the residual activity was measured under standard assay conditions (pH 8.5, 40 °C) at a saturating MAG concentration to determine the residual kcat/Km. The catalytic efficiency after pre-incubation was expressed as a percentage of the efficiency measured for the non-pre-incubated enzyme control.

For the optimal pH determination, the kcat/Km was assessed at 40 °C across a pH range of 5.0 to 9.0 using appropriate buffer systems (e.g., citrate buffer for pH 5.0–6.5, phosphate buffer for pH 6.5–8.0, and Tris-HCl buffer for pH 8.0–9.0). The pH profile was characterized by comparing the kcat/Km values obtained at each pH.

The effect of metal ions on the catalytic efficiency of AOL was investigated by supplementing the standard reaction mixture (50 mM Tris-HCl, pH 8.5, 40 °C) with various metal chlorides or sulfates (e.g., Mg^2+^, Fe^3+^, Fe^2+^, Ca^2+^, Cu^2+^, K^+^, Na^+^, Mn^2+^, Ni^2+^, and Co^2+^) to a final concentration of 1 mM. The kcat/Km was determined in the presence of each metal ion and normalized to the control activity measured in the absence of added metal ions, which was set as 100%.

## 4. Conclusions

This study successfully addressed the critical bottlenecks associated with the heterologous expression and industrial application of *Aspergillus oryzae* lipase (AOL), specifically targeting the challenges of insoluble expression in *Escherichia coli* and limited catalytic efficiency towards monoacylglycerols (MAGs). By integrating fusion tag technology with rational molecular design, we developed a robust biocatalyst, sfGFP-Y92Q, which exhibits superior solubility, catalytic activity, and thermal stability.

The introduction of the sfGFP fusion tag proved effective in overcoming the folding limitations of the wild-type enzyme, significantly enhancing soluble expression and preventing aggregation. Building upon this, rational design guided by computational alanine scanning and saturation mutagenesis identified residue Y92 as a critical site for modulating enzymatic performance. The resulting mutant, sfGFP-Y92Q, demonstrated a remarkable 3.5-fold increase in specific activity compared to the wild type. Furthermore, this mutant exhibited exceptional thermal resilience, with a half-life at 50 °C extended by 2.3-fold, making it highly suitable for industrial processes requiring elevated temperatures.

A pivotal finding of this work is the mutant’s unprecedented efficiency in hydrolyzing MAGs. While the wild-type AOL alone formed inclusion bodies and showed no activity, its soluble sfGFP-fused version achieved a moderate ~65% conversion rate. In contrast, the optimized variant sfGFP-Y92Q reached approximately 98%, significantly outperforming the commercial benchmark Novozym 435 (~54%). Structural analysis suggests that the Y92Q mutation optimizes the active site geometry, likely by enlarging the substrate-binding pocket or altering the local amphiphilic environment, thereby relieving steric hindrance and facilitating the access of bulky MAG substrates.

In summary, this study not only elucidates the structural mechanisms underlying AOL’s substrate specificity and stability but also provides a highly efficient biocatalyst for the oleochemical industry. The sfGFP-Y92Q mutant offers a potent solution for the deep hydrolysis of glycerides, particularly in removing rate-limiting MAG impurities during biodiesel production and oil refining. Its ability to operate efficiently under mild conditions with reduced enzyme dosage underscores its potential for developing low-cost, sustainable, and green biomanufacturing processes. Future work may focus on the scale-up of this biocatalyst and its immobilization to further enhance its industrial viability.

## Data Availability

The original contributions presented in this study are included in the article. Further inquiries can be directed to the corresponding author.

## Supplementary Materials

The following supporting information can be downloaded at: https://www.mdpi.com/article/10.3390/molecules31030398/s1, Table S1: Primersr equired for the experiment.
