# Supplementary material for: Efficient Degradation of Monoacylglycerols by an Engineered Aspergillus oryzae Lipase: Synergistic Effects of sfGFP Fusion and Rational Design
Source: Molecules. 2026 Jan 23;31(3):398. doi: 10.3390/molecules31030398 (PMC12899640; doi:10.3390/molecules31030398)
Supplement: Supplementary file 1 [file molecules-31-00398-s001.zip › molecules-4093686-supplementary.pdf]

Table S1 Primersr equired for the experiment

Primer	SEQUENCE(5'-3')	Application
AOL-1	TACCCGACTGCCATCGACGTCCGCGACATCC CAACTACTCAACTGGAGGACTTCAAGTT	
AOL-2	GTTTGGGCAATAGGTCGCGGCGGCGTACTG AACCCAGAACTTGAAGTCCTCCAGTTGAG	
AOL-3	GCGACCTATTGCCCAAACAACTACGTCGCA AAAGACGGTGAAAAGCTGAACTGCTCTGT	De novo synthesis of the target gene
AOL-4	AGTGGAGCCAGCTGCCTCAACATCCGGGCA GTTACCAACAGAGCAGTTCAGCTTTTCAC	

Primer	SEQUENCE(5'-3')	Application
AOL-5	GGCAGCTGGCTCCACTGTAAAGCTGAGCTTC TCTGACGACACCATCACTGACACTGCTG	
AOL-6	AACTACGATCGCTTTGTTCGTGTTATCTACG GCGACAAAACCAGCAGTGTCAGTGATGG	
AOL-7	CACGAACAAAGCGATCGTAGTTGCATTCCG CGGTTCTTACTCTATCCGTAATTGGGTTA	
AOL-8	CAGGCCAGGGTCGGTTTGAGGAAAGGTCGC ATCGGTAACCCAATTACGGATAGAGTAAG	De novo synthesis of the target gene
AOL-9	CCGACCCTGGCCTGTGCGACGGCTGCAAAG CCGAACTGGGCTTCTGGACCGCCTGGAAA	
AOL-10	TTTCAGCTCATCCAGGGTTTTGATGATACGG TCACGTACTACTTTCCAGGCGGTCCAGA	
AOL-11	CAAAACCCTGGATGAGCTGAAACCGGAACA TTCTGACTACAAAATCGTTGTGGTGGGTC	

AOL-12	CTGCCGCAGCCAGGGATGCGATTGCTGCGC CCAGAGAATGACCCACCACAACGATTTTG	
AOL-13	CCTGGCTGCGGCAGACCTGCGCACCAAGAA CTACGACGCTATCCTGTATGCTTATGCAG	
AOL-14	ATGAATTCCGCCAGCGGTTTGTTCGCAACGC GCGGTGCTGCATAAGCATACAGGATAGC	
AOL-15	CCGCTGGCGGAATTCATCACTAACCAGGGC AACAACTACCGCTTCACTCACAACGACGA	

Primer	SEQUENCE(5'-3')	Application
AOL-16	AACGTAGCCCATGGTCAGCAGCGGCAGTTT CGGTACAGGATCGTCGTTGTGAGTGAAGC	
AOL-17	CTGACCATGGGCTACGTTCACATCTCTCCGG AGTACTACATCACTGCGCCGGACAACAC	
AOL-18	TACATAGCCGTCCAGCACCGTTACCTGGTTA TCGGTTACGGTAGTGTTGTCCGGCGCAG	
AOL-19	GTGCTGGACGGCTATGTAAACTTCAAAGGT AACACCGGCACTAGCGGTGGTCTGCCGGA	De novo synthesis of the target gene
AOL-20	CCGCATGAATGAAATACCAAACGTGGCTGT GGAACGCCAGCAGATCCGGCAGACCACCG	
AOL-21	CGTTTGGTATTTCATTCATGCGGACGCATGC AAAGGCCCAGGCCTGCCTCTGCGT	
AOL-22	ACGCAGAGGCAGGCCTGGGCCTTTGCATGC GT	
PAOL-F	ACGCAGAGGCAGGCCTGGGCCTTTGCATGC GTC	
PAOL-R	TACCCGACTGCCATCGACGTCCGCGACATCC CAACTACTCAACTGGAGG	

$P_{pEt23a\text{-}sfGFP(-15)}$-F-1	CCAGGCCTGCCTCTGCGTTAGTGAGATCCGGCTGCTAACAAAGC	
$P_{pEt23a\text{-}sfGFP(-15)}$-R-1	GTCGATGGCAGTCGGGTATGGACCCTGGAACAAAACCTCCAATTTATAC	

Primer	SEQUENCE(5’-3’)	Application
P_{pEt23a}-F	CCAGGCCTGCCTCTGCGTTAGTGAGATCCGGCTGCTAACAAAG	
P_{pEt23a}-R	GTCGATGGCAGTCGGGTAATGATGATGATGATGGTGCATATGTATATCTCCTTC	
$P_{pEt23a\text{-}sfGFP(-15)}$-F-2	TTGGAGGTTTTGTTCCAGGGTCCAGTGAGCAAG	
$P_{pEt23a\text{-}sfGFP(-15)}$-R-2	GTCGATGGCAGTCGGGTAATGATGATGATGATGGTGCATATGTATATCTCCTTC	
$P\text{-}_{Y29K}$-F	GCGACCAAATGCCCAAACAACTACGTCGCAAAAGACGG	
$P\text{-}_{29K}$-R	TGGGCATTTGGTCGCGGCGGCGTACTGAACCC	
$P\text{-}_{Y29R}$-F	CGCGACCCGCTGCCCAAACAACTACGTCGCAAAAGACGG	
$P\text{-}_{Y29R}$-R	GGGCAGCGGGTCGCGGCGGCGTACTGAACCCAG	AOL point mutation
$P\text{-}_{S91A}$-F	GCGGTGCGTACTCTATCCGTAATTGGGTTACCGATGC	
$P\text{-}_{S91A}$-R	TAGAGTACGCACCGCGGAATGCAACTACGATCGCTTTG	
$P\text{-}_{Y92F}$-F	GGTTCTTTCTCTATCCGTAATTGGGTTACCGATGCGAC	

P-$_{Y92F}$-R	GATAGAGAAAGAACCGCGGAATGCAACTACGATCGC	

Primer	SEQUENCE(5’-3’)	Application
P-$_{Y92H}$-F	GGTTCTCAcTCTATCCGTAATTGGGTTACCGATGCGAC	AOL point mutation
P-$_{Y92H}$-R	GATAGAGTGAGAACCGCGGAATGCAACTACGATCGC	
P-$_{Y92I}$-F	GGTTCTATCTCTATCCGTAATTGGGTTACCGATGCGAC	
P-$_{Y92I}$-R	GATAGAGATAGAACCGCGGAATGCAACTACGATCGC	
P-$_{Y92Q}$-F	GGTTCTCAGTCTATCCGTAATTGGGTTACCGATGCGAC	
P-$_{Y92Q}$-R	GATAGACTGAGAACCGCGGAATGCAACTACGATCGC	
P-$_{S93P}$-F	TCTTACCCTATCCGTAATTGGGTTACCGATGCGACCTTTC	
P-$_{S93P}$-R	ACGGATAGGGTAAGAACCGCGGAATGCAACTACG	
P-$_{N96T}$-F	ATCCGTACCTGGGTTACCGATGCGACCTTTCCTCAAAC	
P-$_{N96T}$-R	AACCCAGGTACGGATAGAGTAAGAACCGCGGAATGCAAC	
P-$_{D100M}$-F	GGGTTACCATGGCGACCTTTCCTCAAACCGACCCTGGC	
P-$_{D100M}$-R	AGGTCGCCATGGTAACCCAATTACGGATAGAGTAAGAACCGCGG	

Primer	SEQUENCE(5’-3’)	Application
P-$_{D100N}$-F	GTTACCAATGCGACCTTTCCTCAAACCGACCCTGGC	
P-$_{D100N}$-R	GGTCGCATTGGTAACCCAATTACGGATAGAGTAAGAACCGCG	
P-$_{D100T}$-F	GTTACCACTGCGACCTTTCCTCAAACCGACCCTGGC	
P-$_{D100T}$-R	GGTCGCAGTGGTAACCCAATTACGGATAGAGTAAGAACCGCG	
P-$_{D100K}$-F	GTTACCAAGGCGACCTTTCCTCAAACCGACCCTGGC	
P-$_{D100K}$-R	GGTCGCCTTGGTAACCCAATTACGGATAGAGTAAGAACCGCG	
P-$_{F103Y}$-F	GCGACCTATCCTCAAACCGACCCTGGCCTG	AOL point mutation
P-$_{F103Y}$-R	TTGAGGATAGGTCGCATCGGTAACCCAATTACGGATAGAGTAAG	
P-$_{F103R}$-F	GCGACCCGCCCTCAAACCGACCCTGGCCTG	
P-$_{F103R}$-R	TTGAGGGCGGGTCGCATCGGTAACCCAATTACGGATAGAGTAAG	
P-$_{F103Q}$-F	GCGACCCAACCTCAAACCGACCCTGGCCTG	
P-$_{F103Q}$-R	TTGAGGTTGGGTCGCATCGGTAACCCAATTACGGATAGAGTAAG	
P-$_{E117Q}$-F	AAAGCCCAGCTGGGCTTCTGGACCGCCTGGAAAGTAG	
P-$_{E117Q}$-R	GCCCAGCTGGGCTTTGCAGCCGTCGCACAGG	

Primer	SEQUENCE(5’-3’)	Application
P-$_{E117R}$-F	AAAGCCTTCCTGGGCTTCTGGACCGCCTGGAAAGTAG	AOL point mutation
P-$_{E117R}$-R	GCCCAGGAAGGCTTTGCAGCCGTCGCACAGG	
P-$_{E117W}$-F	AAAGCCTGGCTGGGCTTCTGGACCGCCTGGAAAGTAG	
P-$_{E117W}$-R	GCCCAGCCAGGCTTTGCAGCCGTCGCACAGG	
P-$_{F120Y}$-F	CTGGGCTATTGGACCGCCTGGAAAGTAGTACGTGACCG	
P-$_{F120Y}$-R	GGTCCAATAGCCCAGTTCGGCTTTGCAGCCGTC	
P-$_{H152N}$-F	GTGGGTAACTCTCTGGGCGCAGCAATCGCATC	
P-$_{H152N}$-R	CAGAGAGTTACCCACCACAACGATTTTGTAGTCAGAATGTTCCG	
P-$_{H152Q}$-F	GTGGGTCAGTCTCTGGGCGCAGCAATCGCATC	
P-$_{H152Q}$-R	CAGAGACTGACCCACCACAACGATTTTGTAGTCAGAATGTTCCG	
P-$_{H152C}$-F	GTGGGTTGCTCTCTGGGCGCAGCAATCGCATC	
P-$_{H152C}$-R	CAGAGAGCAACCCACCACAACGATTTTGTAGTCAGAATGTTCCG	

Primer	SEQUENCE(5’-3’)	Application
P-$_{H152T}$-F	GTGGGTACCTCTCTGGGCGCAGCAATCGCATC	AOL point mutation

P-H152T-R	CAGAGAGGTACCCACCACAACGATTTTGTAGTCAGAATGTTCCG	
P-H152F-F	GTGGGTTTCTCTCTGGGCGCAGCAATCGCATC	
P-H152F-R	CAGAGAGAAACCCACCACAACGATTTTGTAGTCAGAATGTTCCG	
P-L154I-F	CATTCTATCGGCGCAGCAATCGCATCCCTGGCTG	
P-L154I-R	TGCGCCGATAGAATGACCCACCACAACGATTTTGTAGTCAG	
P-L154V-F	CATTCTGTTGGCGCAGCAATCGCATCCCTGGCTG	
P-L154V-R	TGCGCCAACAGAATGACCCACCACAACGATTTTGTAGTCAG	
P-L154W-F	CATTCTTGGGGCGCAGCAATCGCATCCCTGGCTG	
P-L154W-R	TGCGCCCCAAGAATGACCCACCACAACGATTTTGTAGTCAG	
P-P181F-F	GCAGCATTCCGCGTTGCGAACAAACCGCTGGC	
P-P181F-R	AACGCGGAATGCTGCATAAGCATACAGGATAGCGTCGTAGTTC	
P-P181Q-F	GCAGCACAGCGCGTTGCGAACAAACCGCTGGC	

Primer	SEQUENCE(5’-3’)	Application
P-P181Q-R	AACGCGCTGTGCTGCATAAGCATACAGGATAGCGTCGTAGTTC	AOL point mutation

P-P181H-F	GCAGCACACCGCGTTGCGAACAAACCGCTG GC	
P-P181H-R	AACGCGGTGTGCTGCATAAGCATACAGGAT AGCGTCGTAGTTC	
P-P181W-F	GCAGCATGGCGCGTTGCGAACAAACCGCTG GC	
P-P181W-R	AACGCGCCATGCTGCATAAGCATACAGGAT AGCGTCGTAGTTC	
P-P207Y-F	GACGATTACGTACCGAAACTGCCGCTGCTG ACC	
P-P207Y-R	CGGTACGTAATCGTCGTTGTGAGTGAAGCG GTAGTTGTTG	
P-P207R-F	GACGATCGTGTACCGAAACTGCCGCTGCTG ACC	
P-P207R-R	CGGTACACGATCGTCGTTGTGAGTGAAGCG GTAGTTGTTG	
P-P207Q-F	GACGATCAGGTACCGAAACTGCCGCTGCTG ACC	
P-P207Q-R	CGGTACCTGATCGTCGTTGTGAGTGAAGCGG TAGTTGTTG	
P-P207K-F	GACGATAAAGTACCGAAACTGCCGCTGCTG ACC	
P-P207K-R	CGGTACTTTATCGTCGTTGTGAGTGAAGCGG TAGTTGTTG	

Primer	SEQUENCE(5'-3')	Application
P-P207W-F	GACGATTGGGTACCGAAACTGCCGCTGCTG ACC	AOL point mutation

P-P207W-R	CGGTACCCAATCGTCGTTGTGAGTGAAGCG GTAGTTGTTG	
P-V208I-R	TTTCGGAATAGGATCGTCGTTGTGAGTGAAG CGGTAG	
P-V208M-F	GATCCTATGCCGAAACTGCCGCTGCTGACCA TG	
P-V208M-R	TTTCGGCATAGGATCGTCGTTGTGAGTGAAG CGGTAG	
P-V208R-F	GATCCTCGTCCGAAACTGCCGCTGCTGACCA TG	
P-V208R-R	TTTCGGACGAGGATCGTCGTTGTGAGTGAAG CGGTAG	
P-V208Q-F	GATCCTCAGCCGAAACTGCCGCTGCTGACCA TG	
P-V208Q-R	TTTCGGCTGAGGATCGTCGTTGTGAGTGAAG CGGTAG	
P-V208W-F	GATCCTTGGCCGAAACTGCCGCTGCTGACCA TG	
P-V208W-R	TTTCGGCCAAGGATCGTCGTTGTGAGTGAAG CGGTAG	
P-L211R-F	CCGAAACGCCCGCTGCTGACCATGGGCTAC GTTCACATC	
P-L211R-R	CAGCGGGCGTTTCGGTACAGGATCGTCGTTG TGAGTGAAGC	

Primer	SEQUENCE(5’-3’)	Application
P-L211I-F	CCGAAAATCCCGCTGCTGACCATGGGCTAC GTTCACATC	AOL point mutation

P-L211I-R	CAGCGGGATTTTCGGTACAGGATCGTCGTTGTGAGTGAAGC	
P-P212S-F	AAACTGTCTCTGCTGACCATGGGCTACGTTCACATCTC	
P-P212S-R	CAGCAGAGACAGTTTCGGTACAGGATCGTCGTTGTGAG	
P-L213W-F	CTGCCGTGGCTGACCATGGGCTACGTTCACATCTCTCC	
P-L213W-R	GGTCAGCCACGGCAGTTTCGGTACAGGATCGTCGTTGTG	
P-M216R-F	CTGACCCGTGGCTACGTTCACATCTCTCCGGAGTACTACATCAC	
P-M216R-R	GTAGCCACGGGTCAGCAGCGGCAGTTTCGGTACAG	
P-M216W-F	CTGACCTGGGGCTACGTTCACATCTCTCCGGAGTACTACATCAC	
P-M216W-R	GTAGCCCCAGGTCAGCAGCGGCAGTTTCGGTACAG	
P-M216Y-F	CTGACCTACGGCTACGTTCACATCTCTCCGGAGTACTACATCAC	
P-M216Y-R	GTAGCCGTAGGTCAGCAGCGGCAGTTTCGGTACAG	

Primer	SEQUENCE(5’-3’)	Application
P-F265R-F	CTGGCGCGTCACAGCCACGTTTGGTATTTCATTCATGCGGAC	AOL point mutation
P-F265R-R	GCTGTGACGCGCCAGCAGATCCGGCAGACCACCG	

P-F265W-F	CTGGCGTGGCACAGCCACGTTTGGTATTTCA TTCATGCGGAC	
P-F265W-R	GCTGTGCCACGCCAGCAGATCCGGCAGACC ACCG	
P-V269D-F	AGCCACGATTGGTATTTCATTCATGCGGACG CATGCAAAG	
P-V269D-R	ATACCAATCGTGGCTGTGGAACGCCAGCAG ATCCG	
P-D276N-F	CATGCGAACGCATGCAAAGGCCCAGGCCTG C	
P-D276N-R	GCATGCGTTCGCATGAATGAAATACCAAAC GTGGCTGTGG	
P-D276Q-F	CATGCGCAGGCATGCAAAGGCCCAGGCCTG C	
P-D276Q-R	GCATGCCTGCGCATGAATGAAATACCAAAC GTGGCTGTGG	
P-D276H-F	CATGCGCACGCATGCAAAGGCCCAGGCCTG C	
P-D276H-R	GCATGCGTGCGCATGAATGAAATACCAAAC GTGGCTGTGG	
P-D276K-F	CATGCGAAAGCATGCAAAGGCCCAGGCCTG C	

Primer	SEQUENCE(5’-3’)	Application
P-D276K-R	GCATGCTTTCGCATGAATGAAATACCAAAC GTGGCTGTGG	AOL point mutation
P-D276S-F	CATGCGAGCGCATGCAAAGGCCCAGGCCTG C	

P-$_{D276S}$-R	GCATGCGCTCGCATGAATGAAATACCAAACGTGGCTGTGG
P-$_{D276W}$-F	CATGCGTGGGCATGCAAAGGCCCAGGCCTGC
P-$_{D276W}$-R	GCATGCCCACGCATGAATGAAATACCAAACGTGGCTGTGG
P-$_{K279F}$-F	GCATGCTTCGGCCCAGGCCTGCCTCTGCGTTAG
P-$_{K279F}$-R	TGGGCCGAAGCATGCGTCCGCATGAATGAAATACCAAACGTG
P-$_{K279W}$-F	GCATGCTGGGGCCCAGGCCTGCCTCTGCGTTAG
P-$_{K279W}$-R	TGGGCCCCAGCATGCGTCCGCATGAATGAAATACCAAACGTG
